# Knowledge and perceptions of the midwifery profession among university students in Luxembourg

**DOI:** 10.3389/fgwh.2026.1846983

**Published:** 2026-06-10

**Authors:** Joeri Vermeulen, Maaike Fobelets, Annabelle Pierron, Christian Grévisse, Anna-Cristina Alborino-Rings, Kristel von Laufenberg, Thanh-Van Trieu, Ronald Buyl, Ali Ghanchi

**Affiliations:** 1 Luxembourg Midwifery Innovations in Research and Education (LUMIRE) Research Group, Department of Health, Medicine and Life Sciences, Faculty of Science, Technology and Medicine, University of Luxembourg, Esch-sur-Alzette, Grand Duchy of Luxembourg; 2 Department of Public Health, Faculty of Medicine and Pharmacy, Vrije Universiteit Brussel (VUB), Brussels, Belgium; 3 Association Luxembourgeoise des Sages-Femmes, Strassen, Luxembourg; 4 Centre Hospitalier de Luxembourg, Luxembourg City, Luxembourg

**Keywords:** health education, maternity care models, midwifery, midwifery practice, public perceptions

## Abstract

**Introduction:**

Knowledge of midwives’ competencies and preferences for maternity care providers play an important role in shaping care-seeking behavior and the utilization of midwifery-led models of care. However, limited research has explored these aspects among young adults, particularly in multicultural contexts such as Luxembourg.

**Methods:**

A cross-sectional study was conducted among university students in Luxembourg using an online survey based on the validated Midwifery Profiling Questionnaire (MidProQ). The questionnaire assessed knowledge of midwives’ legal competencies during pregnancy, labor, and childbirth, as well as preferred and perceived central maternity care providers. Descriptive statistics and chi-square tests were used to examine associations between knowledge and sociodemographic characteristics.

**Results:**

A total of 133 participants were included, the majority being female (75.9%) and aged 18–24 years (60.2%). Knowledge of midwives’ legal competencies varied significantly by gender, age, language background, and educational level. Female participants and those enrolled in advanced or health-related programs demonstrated higher levels of agreement with midwives’ competencies. Preferences for maternity care providers were stage-specific: obstetricians were predominantly preferred and perceived as central during pregnancy, whereas midwives were more frequently preferred and perceived as central during labor and childbirth.

**Discussion:**

University students in Luxembourg demonstrate knowledge of midwives’ legal competencies that varies significantly by gender, age, language background, and educational level, alongside stage-specific preferences for maternity care providers. These findings highlight the need for targeted, culturally sensitive educational and policy strategies to improve public understanding of midwives’ roles. Strengthening awareness may support informed decision-making, promote appropriate utilization of midwifery-led care, and contribute to workforce sustainability and the development of midwifery-led models of care in Luxembourg and similar multicultural settings.

## 1 Introduction

Despite the plethora of studies examining the provision of maternity services, there remains a notable paucity of research exploring maternity care from women's perspectives—particularly those of young women and their partners. Specifically, limited attention has been paid to their preferences, knowledge, and opinions regarding midwifery, maternity care, and normal labor and childbirth (1, 2). Furthermore, studies conducted in the United States and Belgium have reported that women—especially nulliparous women in early adulthood—often lack adequate information about available maternity care options and the competencies of midwives (3, 4). It has been well established that women's choices regarding maternity care are shaped by the organization of services, the influence of care providers, as well as cultural, psychosocial, and demographic factors (5–7). With the current shift toward women-centered care, understanding individuals’ preferences for healthcare providers during childbirth has become increasingly important for policymakers and midwifery advocates (8). However, international research specifically exploring the knowledge and preferences of young adults regarding midwifery care remains limited, and data from Luxembourg are lacking. This study addresses this gap by examining university students as future users of maternity services within a multilingual and multicultural European context.

Luxembourg is a highly multicultural country with a diverse population comprising more than 112 nationalities. In addition, Luxembourgish, French and German are the national languages, and all three languages play an important role in everyday communication and identity. Luxembourgish remains the most widely spoken language in Luxembourg, which may also shape how health information and maternity care are perceived and accessed. To our knowledge, this is one of the first European studies exploring these issues among young, educated individuals—such as university students—who represent future users of maternity care services. This study therefore provides a first exploratory insight into the Luxembourgish student population.

In the Grand Duchy of Luxembourg, midwives are autonomous and are a regulated profession, qualified to autonomously manage normal pregnancy, labor and childbirth (9). Consequently, midwives have diagnostic and prescriptive authority. This level of autonomy may be considered relatively advanced within the European context, where midwives’ scope of practice is variably defined and, in some contexts, more closely integrated within physician-led models of care (10). To ensure that midwives have the necessary competencies for the full scope of professional practice, midwifery education in Luxembourg consists of a 4-year undergraduate university bachelor (11). In addition to clinical competencies, the development of emotional intelligence and critical thinking is essential for fostering clinical judgment, a key component of autonomous midwifery practice (12). A 2019 study conducted across Europe revealed significant underutilization of midwives, whose professional responsibilities are often limited in practice (10). Similarly, in Luxembourg, midwives are not consistently fulfilling their autonomous roles, as most pregnant women continue to receive care from obstetricians (13). The situation is further complicated by the fact that the roles and responsibilities of midwives are not widely understood by the public. This lack of clarity is compounded by the growing presence of non-clinical birth support providers, such as doulas and birthing coaches, who—while not healthcare professionals—may be perceived as alternatives to midwifery care (14). Public understanding is further challenged in Luxembourg by its highly international population, as perceptions of midwifery and the organization of maternity care vary considerably across countries. Additionally, a national shortage of midwives exacerbates the issue (13).

A comprehensive assessment of young people's perceptions of midwifery competencies is particularly important, as they represent the next generation of users of maternity services. Understanding their level of knowledge can reveal gaps that influence care-seeking behavior, shape the utilization of midwifery services, and ultimately affect maternal and newborn outcomes (15). Furthermore, as public recognition is fundamental to professional status, it is essential that both women and men in Luxembourg understand and value the role of midwives. Assessing public knowledge of the midwifery profession therefore provides a critical foundation for targeted educational initiatives, informed policy development, and alignment with population needs and healthcare system priorities.

### 1.1 Aims

This study aims to explore the level of knowledge and the perceptions held by university students in Luxembourg regarding the midwifery profession, with a particular focus on midwives’ legal competencies and their role in providing care during normal pregnancy, labour, and childbirth, as measured by the MidProQ questionnaire (14). Specifically, the study seeks to assess students’ knowledge of midwives’ legal competencies across pregnancy, labor, and childbirth, to identify sociodemographic and educational factors associated with variations in knowledge levels, and to describe students’ preferences and perceived central maternity care providers across the maternity care continuum.

## 2 Material and methods

### 2.1 Study design, sample and setting

A cross-sectional study using a convenience sample of University of Luxembourg students was conducted to recruit participants. Data collection took place between October and December 2025. Recruitment was conducted through a targeted announcement on the university's internal online learning platform, inviting students to participate in the online survey. A convenience sampling approach was chosen due to its feasibility and the ease of access to a large and diverse university population within the study timeframe; however, this approach may limit the generalizability of the findings.

### 2.2 Inclusion and exclusion criteria

Participants were required to be aged 18 years or older and enrolled in a degree-granting program at the University of Luxembourg. Individuals were excluded if they were under 18 years of age, staff members of the University, midwifery students, or students attending courses at the University without being enrolled in a University of Luxembourg degree program (e.g., students from the National School of Health of Luxembourg — École nationale de santé du Luxembourg —, Competence Centre Management Certificate programs, or other continuing education initiatives). Midwifery students were excluded because their specialized training could bias baseline perceptions. Students not enrolled in a University of Luxembourg degree program were excluded to maintain sample homogeneity and reflect perceptions of the core universities’ student body.

### 2.3 Data collection

Data were collected using an exploratory design through an online semi-structured survey (Qualtrics^TM^) that included both open- and closed-ended questions. The questionnaire was based on the validated Midwifery Profiling Questionnaire (MidProQ) to assess public awareness of midwives’ roles and competencies (16), originally designed and used by a Belgian research team (4). The questionnaire assesses knowledge of midwives’ legal competencies during pregnancy (19 questions), labor (8 questions), and childbirth (8 questions), and includes single-choice questions assessing preferred and perceived central care providers for each stage. Socio-demographic characteristics were also collected.

As the University of Luxembourg is a multilingual institution with English, French, and German as its official languages, the MidProQ was distributed in all three languages for the present study. It was independently translated from English into the two additional languages by three multilingual researchers who were native language speaker of the translated languages (AP, KVL, AA). Following best practices in the literature, all language versions were reviewed by the multilingual research team to ensure conceptual equivalence (17). Minor linguistic amendments were made to improve clarity and consistency of wording across languages without altering the meaning of the items. Back-translation and formal pilot testing were not conducted, which may have limited the assessment of cross-language equivalence and participant comprehension.

### 2.4 Data analysis

Descriptive statistics (frequencies and percentages) were calculated to summarize participants’ socio-demographic characteristics, their agreement with midwives’ legal competencies across pregnancy, labor, and childbirth, and their preferences and perceptions regarding the central maternity care provider.

Associations between participants’ agreement with midwives’ legal competencies and socio-demographic variables (gender, age group, Luxembourgish language background, first-hand experience of pregnancy or childbirth, and university program) were examined using Chi-square (*χ*^2^) tests. Statistical significance was defined as *p* < 0.05. Given the exploratory nature of this study, no adjustment for multiple comparisons (e.g., Bonferroni correction) was applied; therefore, findings should be interpreted with caution due to the increased risk of Type I error. Data were analyzed using IBM SPSS Statistics version 31.0.0.

### 2.5 Ethics

Ethical approval was obtained from the Ethics Review Panel of the University of Luxembourg in September 2025 (ERP 25-087 MidProQ-LUX). Informed consent from the participants was gained through their agreement to participate on the first page of survey, with completion of the inquiry also indicating their willingness to take part. At any time, participants could decide not to answer a question or withdraw from the study.

## 3 Results

Most participants (*n* = 133, 75.9%) identified as female, while 20.3% identified as male. A small proportion identified as non-binary (*n* = 3, 2.3%) or preferred not to disclose their gender (*n* = 2, 1.5%). Most respondents were aged 18–24 years (60.2%), followed by those aged 25–29 years (21.8%) and 30 years or older (18.0%). Regarding reproductive experience, most participants reported no first-hand experience of pregnancy, either personally or through a partner (86.5%). Similarly, 89.5% indicated that neither they nor their partner had experienced childbirth.

Most respondents were enrolled in a Bachelor's program (60.6%), followed by Master's (23.5%) and Doctoral programs (15.2%). In terms of faculty affiliation, nearly half of the participants were enrolled in Humanities, Education, and Social Sciences (48.5%), followed by Science, Technology, and Medicine (29.5%) and Law, Economics, and Finance (20.5%) (Table 1).

**Table 1:** Sociodemographic characteristics.

Characteristic	Category	N	%
Gender	Female	101	75,9%
	Male	27	20,3%
	Non-Binary or Prefer not to say	5	3,8%
Age group	18–24 Years	80	60,2%
	25–29 Years	29	21,8%
	30 + Years	24	18,0%
First-hand experience of pregnancy (self or partner)	No	115	86,5%
	Yes	18	13,5%
First-hand experience of childbirth (self or partner)	No	119	89,5%
	Yes	14	10,5%
University program	Bachelor	80	60,6%
	Master	31	23,5%
	Doctorate	20	15,2%
	Other	1	0,8%
Faculty	Humanities, Education and Social Sciences	64	48,5%
	Science, Technology and Medicine	39	29,5%
	Law, Economics and Finance	27	20,5%
	Other	2	1,5%

### 3.1 Participant's knowledge of midwives’ legal competencies

#### 3.1.1 During pregnancy

Overall, participants showed greater agreement with midwives’ involvement in core aspects of antenatal care, such as monitoring uncomplicated pregnancies, providing information, and supporting preparation for childbirth. In contrast, lower agreement was observed for more specialized or medicalized competencies, including performing ultrasounds, prescribing examinations, or conducting invasive procedures such as amniocentesis or abortion.

Gender differences were observed for several pregnancy-related competencies. Agreement that midwives may autonomously apply fetal heart rate monitoring, support preparation for childbirth, perform vaginal examinations, measure maternal blood pressure, conduct antenatal classes, provide yoga classes, and deliver nutrition counselling differed significantly between female and male participants (all *p* ≤ 0.040), with women generally reporting higher agreement for these activities. Across all significant items, female participants consistently reported higher agreement than male participants.

Age-related differences were identified for performing vaginal examinations, taking vaginal swab tests, and measuring blood pressure (*p* ≤ 0.048), with higher levels of agreement generally observed among participants aged 25–29 years and ≥30 years compared to those aged 18–24 years. Agreement also differed significantly by language background. Participants with a Luxembourgish language background showed item-specific variations in agreement regarding midwives’ autonomy to diagnose pregnancy, prescribe diagnostic examinations, perform ultrasounds, perform abortions, and administer vaccinations during pregnancy (all *p* ≤ 0.024), with some items showing higher agreement and others lower agreement compared to participants with other language backgrounds.

Differences were also observed according to university program. Agreement regarding several pregnancy-related competencies differed significantly between students enrolled in Bachelor's, Master's, and Doctoral programs (all *p* ≤ 0.036), with students in Master's and Doctoral programs generally reporting higher agreement than those in Bachelor's programs. First-hand experience of pregnancy or childbirth showed fewer associations. However, participants with prior pregnancy or childbirth experience differed significantly in their agreement regarding blood pressure measurement and abdominal palpation during pregnancy (*p* ≤ 0.031).

#### 3.1.2 During labor and childbirth

During labor, participants generally recognized midwives’ role in supporting normal labor, including monitoring its progression and providing non-medical pain relief. However, agreement was lower for more medicalized interventions, such as administering medication for labor stimulation or performing procedures like artificial rupture of membranes.

During labor, agreement that midwives may autonomously follow up an uncomplicated labor differed by gender, language background, and university program (all *p* ≤ 0.030). Female participants and those enrolled in health-related or advanced degree programs generally expressed higher agreement. Gender differences were also observed for agreement regarding vaginal examinations during labor (*p* = 0.007). Participants’ language background was significantly associated with agreement regarding the administration of medication to stimulate contractions and the provision of non-medical pain relief methods (both *p* = 0.004), suggesting differing perceptions of midwives’ scope of practice across linguistic groups. Agreement regarding non-medical pain relief methods also differed significantly by university program (*p* < 0.001). Participants with first-hand experience of pregnancy or childbirth reported significantly different levels of agreement regarding the artificial rupture of membranes (both *p* < 0.001).

Fewer differences were observed during childbirth. Agreement regarding the use of forceps and the performance of a Caesarean section differed significantly by language background (*p* ≤ 0.012). For most other childbirth-related actions, including uncomplicated delivery, suturing, newborn examination, and maternal or neonatal resuscitation, no significant differences were found across gender, age, reproductive experience, or university program (Table 2).

**Table 2:** Participants’ agreement with midwives’ legal scope of practice across pregnancy, labor, and childbirth.

Midwives' legal competencies	Gender	Age group	Luxembourgish as a most spoken language^a^	First-hand experience of pregnancy (self or partner)	First-hand experience of childbirth (self or partner)	University program
During pregnancy						
Follow up an uncomplicated pregnancy	0.292	0.210	0.835	0.819	0.504	**0**.**027**
Diagnose the pregnancy (via blood or urine sample)	0.219	0.299	**0**.**024**	0.344	0.952	0.444
Inform about the progression of the pregnancy	0.288	0.663	0.841	0.323	0.504	0.060
Listen to the fetal heartbeat	0.641	0.864	0.388	0.115	0.085	0.171
Apply a monitoring to register the fetal heartbeat and the contractions	**0**.**018**	0.268	0.374	0.229	0.532	0.315
Supporting the pregnant woman in her preparation for childbirth (exercises, breathing techniques, etc.)	**<0**.**01**	0.064	0.464	0.129	0.067	**<0**.**001**
Perform a vaginal examination to assess the dilation of the cervix	**0**.**007**	**0**.**047**	0.367	0.942	0.893	0.229
Prescribe exams which are necessary to monitor the progression of the pregnancy (e.g., ultrasound, take a blood sample)	0.246	0.621	**0**.**013**	0.984	0.393	0.707
Take a vaginal swab test	0.569	**0**.**048**	**0**.**002**	0.375	0.811	0.264
Perform an ultrasound	0.621	0.473	**<0**.**001**	0.963	0.971	**0**.**003**
Perform an abortion	0.616	0.738	**0**.**001**	0.292	0.327	0.074
Perform an amniocentesis	0.296	0.761	0.082	0.208	0.110	0.832
Check if the membranes are ruptured (when that is presumed)	0.755	0.606	0.213	0.939	0.952	0.622
Perform a blood sample	0.223	0.921	0.222	0.321	0.830	0.305
Interpret blood results related to the pregnancy	0.521	0.848	0.474	0.480	0.766	0.515
Prescribe medication which are related to the pregnancy	0.779	0.837	0.090	0.940	0.753	0.878
Vaccinate the pregnant woman according to medical recommendations	0.256	0.339	**0**.**003**	0.819	0.487	0.847
Regular weight checks to help track whether the pregnant woman is gaining weight within recommended limits and to prevent complications	0.088	0.460	0.856	0.430	0.236	**0**.**003**
Measure the pregnant woman's blood pressure to detect the early onset of complications	**0**.**013**	**0**.**019**	0.753	**0**.**008**	**0**.**001**	**<0**.**001**
Perform antenatal classes	**<0**.**001**	0.288	0.303	0.516	0.253	**0**.**036**
Provide yoga classes to pregnant women	**0**.**040**	0.792	0.669	0.368	0.318	0.051
Perform acupuncture for the pregnant woman — during her pregnancy, during labor, and after birth	0.059	0.852	0.434	0.188	0.399	0.245
Antenatal classes in a swimming pool	0.350	0.961	0.408	0.942	0.893	0.058
Prepare the couple for parenthood	0.068	0.498	0.670	0.819	0.859	**0**.**013**
Perform nutrition counselling	0.008	0.613	0.273	0.570	0.471	0.232
Palpation of the mother's abdomen to determine the position of the baby in the womb	0.061	0.191	0.447	0.028	**0**.**031**	0.071
During labor						
Follow up an uncomplicated labor	0.030	0.578	**0**.**025**	0.361	0.628	**0**.**019**
Listen to the fetal heartbeat	0.053	0.202	0.712	0.971	0.915	0.292
The early recognition of deviations from the normal course of labor or delivery, including screening for pathology and complications	0.054	0.739	0.219	0.922	0.751	0.292
Apply a monitoring to register the fetal heartbeat and the contractions	0.715	0.721	0.056	0.784	0.731	0.247
Perform a vaginal examination	**0**.**007**	0.334	0.560	0.236	0.274	0.508
Artificially rupture the membranes	0.584	0.084	0.821	**<0**.**001**	**<0**.**001**	0.378
Administer medication for the stimulation of contractions	0.071	0.123	**0**.**004**	0.251	0.170	0.451
Administer an epidural anesthesia for pain relief during labor	0.243	0.951	0.198	0.128	0.209	0.691
Provide non-medical pain relief methods (e.g., massage, relaxation, breathing techniques)	0.058	0.617	**0**.**004**	0.430	0.236	**<0**.**001**
During childbirth						
Perform an uncomplicated delivery	0.562	0.869	0.273	0.620	0.728	0.129
Cut the umbilical cord	0.153	0.467	0.339	0.827	0.554	0.068
Perform an episiotomy at childbirth	0.411	0.259	0.787	0.338	0.072	0.673
Suture an episiotomy after childbirth	0.350	0.669	0.601	0.522	0.373	0.641
Suture a tear after childbirth	0.806	0.953	0.339	0.426	0.303	0.883
Perform a vacuum extraction	0.889	0.226	0.149	0.153	0.270	0.453
Perform a forceps	0.348	0.249	**0**.**012**	0.274	0.714	0.206
Perform a Caesarean Section	0.851	0.246	**<0**.**001**	0.509	0.517	0.160
Examining the newborn to assess their general health and detect any possible abnormalities at birth	0.438	0.900	0.161	0.758	0.851	0.214
Resuscitate the newborn in case of distress at birth, according to neonatal resuscitation protocols	0.574	0.628	0.204	0.817	0.306	0.382
Resuscitate the mother in case of a complication related to childbirth, according to obstetric emergency protocols	0.946	0.415	0.693	0.178	0.675	0.514

*P* = Chi^2^ test.

a Participants could report multiple most spoken languages (French, German, Luxembourgish, Spanish, Portuguese, other).

Bold values indicate statistically significant associations (Chi-square test, *p* < 0.05).

### 3.2 Participants’ preferences and perceived central care providers in normal pregnancy, labor, and childbirth

Nearly half of the participants preferred an obstetrician for the follow-up of a normal pregnancy (49.3%), while one third preferred a midwife (33.6%). Smaller proportions preferred a general practitioner (6.0%) or were unsure about their preference (6.0%). For the follow-up of a normal labor, most participants preferred a midwife (52.2%). An obstetrician was preferred by 26.1% of respondents. A notable proportion of participants reported being uncertain about their preference (11.2%). When asked about preferences for normal childbirth, midwives were most frequently preferred (44.0%), followed by obstetricians (35.1%). Smaller proportions preferred other providers or reported uncertainty (6.7%).

More than half of the participants considered an obstetrician to be the central caregiver for the follow-up of a normal pregnancy (56.4%), while 31.6% identified a midwife as the central caregiver. For normal labor, participants most perceived the midwife as the central caregiver (50.4%). An obstetrician was identified as central by 31.6% of respondents, while 8.3% were unsure. In the context of childbirth, the midwife was most frequently perceived as the central caregiver (42.9%), followed by the obstetrician (33.8%). A smaller proportion identified a general practitioner (11.3%) or reported uncertainty (6.8%). Overall, ‘Don't know’ responses were reported by 6.0%, 11.2%, and 6.7% of participants for preferences regarding pregnancy, labor, and childbirth, respectively, and by 3.0%, 8.3%, and 6.8% for perceptions of the central caregiver (Table 3).

**Table 3:** Participants’ preferences and perceived central care providers in normal pregnancy, labor, and childbirth.

Stage and measure	Care provider	N	%
Pregnancy (preference)	Midwife	45	33,6%
	Obstetrician	66	49,3%
	General Practitioner	8	6,0%
	Nurse	1	0,7%
	Doula	6	4,5%
	Don't know	8	6,0%
Labor (preference)	Midwife	70	52,2%
	Obstetrician	35	26,1%
	General Practitioner	6	4,5%
	Nurse	8	6,0%
	Doula	0	0,0%
	Don't know	15	11,2%
Childbirth (preference)	Midwife	59	44,0%
	Obstetrician	47	35,1%
	General Practitioner	8	6,0%
	Nurse	8	6,0%
	Doula	3	2,2%
	Don't know	9	6,7%
Pregnancy (central caregiver)	Midwife	42	31,6%
	Obstetrician	75	56,4%
	General Practitioner	8	6,0%
	Nurse	1	0,8%
	Doula	3	2,3%
	Don't know	4	3,0%
Labor (central caregiver)	Midwife	67	50,4%
	Obstetrician	42	31,6%
	General Practitioner	7	5,3%
	Nurse	6	4,5%
	Doula	0	0,0%
	Don't know	11	8,3%
Childbirth (central caregiver)	Midwife	57	42,9%
	Obstetrician	45	33,8%
	General Practitioner	15	11,3%
	Nurse	6	4,5%
	Doula	1	0,8%
	Don't know	9	6,8%

*N*, number of participants. Percentages are calculated within each stage (row percentages) and sum to approximately 100% per stage. Minor discrepancies are due to rounding.

## 4 Discussion

To our knowledge, this study is the first to examine participants’ knowledge of midwives’ legal competencies alongside their preferences and perceptions of central care providers across normal pregnancy, labor, and childbirth within the Luxembourgish context, and is among the first studies to explore these aspects within the broader EU context. Overall, the findings demonstrate that knowledge of midwives’ legal scope of practice is uneven and strongly shaped by sociodemographic characteristics (such as gender, age, and language background) as well as educational factors, rather than being uniformly shared.

The findings further indicate that gender, language background, and educational level emerged as key determinants influencing how midwives’ competencies are understood. Female participants and those enrolled in more advanced or health-related university programs demonstrated broader and more accurate knowledge, particularly regarding routine clinical, preventive, and supportive tasks. Similar patterns have been reported elsewhere in Europe. In Poland, women's knowledge of midwives’ competencies was limited and often confused with doctors’ roles, although higher education and greater contact with midwives were associated with better understanding (18). In Slovenia, midwives were widely recognized for their role in labor and breastfeeding, yet fewer women believed they could independently manage an uncomplicated pregnancy, frequently favoring obstetricians (19). A study conducted in Brussels using the same MidProQ instrument also reported limited knowledge of midwives’ legal competencies, but showed a stronger preference for obstetricians and a more limited recognition of midwives’ central role compared to our findings (4). In contrast, our data suggest that personal experience of pregnancy or childbirth alone may not necessarily lead to an accurate understanding of midwives’ legal roles. This may reflect that even when individuals have contact with maternity services, the roles and responsibilities of different providers are not always explicitly explained. Similar findings were reported in Brussels, where knowledge of midwives’ legal competencies remained limited even among women who had previously given birth (4).

Language-related differences highlight the challenges posed by Luxembourg's cultural and linguistic diversity. However, these differences may not solely reflect language itself, but also the country's highly diverse expatriate population. In Luxembourg, language background is closely intertwined with nationality, migration history, and prior exposure to different maternity care systems. As a result, participants’ perceptions of midwives’ roles may reflect norms and experiences from their countries of origin rather than language alone. A recent systematic review highlights the scarcity of tailored health-literacy interventions for pregnant women with limited language proficiency and calls for co-designed, multilingual programs to reduce inequities (20). Language barriers and low health literacy can limit understanding of available maternity roles and services, particularly in linguistically diverse populations (21). However, these findings should be interpreted in relation to the present study population. Participants in this study were university students, representing a relatively highly educated group, many of whom may be considered expatriates rather than migrants in the traditional sense. In this context, differences in understanding may be less related to low health literacy and more to limited familiarity with the Luxembourgish healthcare system. As such, participants’ perceptions of midwives’ roles may reflect prior experiences and healthcare models from their countries of origin, as well as potential confusion between professional roles, such as those of midwives and obstetricians. This underscores the importance of culturally sensitive and context-specific communication strategies to improve public understanding of midwives’ competencies, even among highly educated and internationally mobile populations. Multilingual educational materials (in Luxembourgish, French, German, and Portuguese) should be developed and disseminated through university health services and public maternity clinics.

Across the maternity care continuum, a clear shift was observed in both preferences and perceptions of the central caregiver. Obstetricians were predominantly preferred and perceived as central during pregnancy, reflecting a medicalized view of this phase. However, as care progressed toward labor and childbirth, midwives increasingly emerged as both the preferred provider and the professional perceived as central. This pattern suggests that women distinguish between stages of maternity care, associating obstetricians with medical oversight in pregnancy and midwives with normal birth, continuous support, and hands-on care during labor and childbirth. The relatively smaller number of subgroup differences observed during childbirth may reflect participants’ more limited familiarity with midwives’ roles during the delivery phase itself, compared to earlier stages of care. These findings are consistent with the existing literature, which shows that across diverse settings women experience and describe maternity care in stage-specific ways, typically linking obstetricians to medical oversight and risk, and midwives to normal birth, continuous presence, and person-centered, hands-on support during labor and birth (22–24).

Our findings have several implications for practice and policy. First, clearer public information is needed to delineate midwives’ legal competencies, particularly during pregnancy and labor, where uncertainty is greatest. Educational initiatives should be tailored to different demographic and linguistic groups to ensure equitable access to accurate information (20). For example, a mandatory module on maternity care options and professional roles could be integrated into the university's general health education curriculum, or an infographic campaign could be displayed in university health centers and student housing. Second, interprofessional collaboration and consistent messaging between obstetricians, pediatricians and midwives may help reduce role ambiguity and strengthen trust in midwifery-led care (25–27). Finally, acknowledging women's differentiated preferences across the maternity continuum can support the design of care models that better align professional roles with women's expectations, potentially improving satisfaction, continuity of care, and appropriate use of maternity services (28).

In the context of workforce shortages and ongoing efforts to strengthen healthcare professions in Luxembourg, improving public understanding of midwives’ competencies is particularly relevant (10). Recruitment challenges—especially among non-Luxembourgish students due to linguistic requirements—underscore the importance of clearly communicating the scope, value, and career opportunities of midwifery. Strengthening public awareness may support recruitment and retention efforts, enhance recognition of midwives’ expertise, and contribute to improved access to safe and respectful maternity care, in line with national and WHO/Europe priorities (18, 29, 30).

### 4.1 Strengths and limitations

This study is the first to examine knowledge of midwives’ legal competencies alongside preferences and perceptions of central maternity care providers across pregnancy, labor, and childbirth in Luxembourg. Its continuum-based approach captures stage-specific differences in professional roles, offering nuanced insight into how perceptions shift across the maternity care pathway. By identifying gender, educational level, and language background as key determinants of knowledge, the findings provide an important basis for targeted educational strategies and policy interventions relevant to both maternity care delivery and workforce planning and contribute evidence on sociocultural barriers and enabling conditions for the implementation and leadership of continuous midwife-led models of care.

However, the study is limited by its focus on university students, who may not be representative of the broader population of maternity service users. In particular, as data were collected from a single university, the findings may not generalize to students in other Luxembourgish educational settings, such as vocational training or non-university higher education institutions. The cross-sectional design precludes causal inference, and reliance on self-reported knowledge may overestimate actual understanding of midwives’ legal competencies and may be subject to social desirability bias, with participants potentially providing responses they perceived as correct. Additionally, although the MidProQ instrument has been previously validated, it was not specifically pilot tested in the Luxembourgish context, which may affect the reliability or interpretation of some items. Additionally, while language differences were identified, the complexity of Luxembourg's multilingual health communication context may not be fully captured. Moreover, no data were collected on participants’ nationality or length of residence in Luxembourg; as such, language background may only partially reflect migration history and familiarity with the healthcare system. Furthermore, although midwifery students were excluded, information on participants’ personal or family background in midwifery or obstetrics (e.g., having a relative working in the field) was not collected, which may have influenced their level of knowledge and perceptions. Nevertheless, Luxembourg's highly diverse population may provide insights that are relevant for other multicultural settings. The findings may therefore be transferable to other countries and contexts, particularly those facing healthcare workforce shortages, where understanding perceptions of university students could help inform recruitment strategies and workforce planning.

Future research should include more diverse population groups beyond university students to enhance generalizability and increase heterogeneity within the sampled population. In particular, the relatively small sample size and the age distribution of the current sample—where around 60% of participants were between 18 and 25 years old—should be considered, and future studies could explore whether the observed findings are primarily related to age or to educational and occupational status (e.g., university students compared with school leavers of the same age). Longitudinal, interventional, and qualitative studies involving women, men, and healthcare professionals are needed to examine how knowledge of midwives’ legal competencies develops over time and to better understand the mechanisms underlying role ambiguity and misunderstandings, particularly within Luxembourg's multilingual context. For example, a randomized controlled trial could test the effect of a brief educational video on students’ knowledge of midwives’ competencies. Comparative studies across countries or care models could further contextualize these findings and identify transferable strategies to strengthen public understanding and utilization of midwifery-led care, particularly in multicultural settings such as Switzerland, Canada, and Belgium.

## 5 Conclusion

This study provides novel insight into university students’ knowledge of midwives’ legal competencies and their preferences for maternity care providers in Luxembourg. The findings demonstrate that knowledge is uneven and influenced by sociodemographic and educational factors, with notable differences according to gender, language background, and level of study, and that students exhibit stage-specific preferences for maternity care providers. While midwives are increasingly recognized as central providers during labor and childbirth, obstetricians remain predominantly associated with pregnancy care, reflecting a persistent medicalized perception of this stage.

These findings highlight the need for clearer, targeted, and culturally sensitive communication strategies to improve public understanding of midwives’ roles, particularly in multilingual and internationally diverse contexts such as Luxembourg. Strengthening awareness of midwives’ competencies and implementing targeted educational and policy measures may support informed decision-making, promote appropriate utilization of midwifery-led care, enhance interprofessional collaboration, and contribute to workforce sustainability and the strengthening of midwifery-led models of care within the Luxembourgish maternity system. A potential next step could involve collaborative efforts to co-develop a publicly available, multilingual digital resource that clarifies midwives’ legal competencies across the maternity care continuum.

## Data Availability

The raw data supporting the conclusions of this article will be made available by the authors, without undue reservation.
